# Book Notices

**Published:** 1889-02

**Authors:** 


Annual of the Universal Medical Sciences. A yearly report of the progress of the general sanitary sciences throughout the world, edited by Chas. E. Sajous, M. D., Philadelphia, and 70 associate editors. Assisted by over two hundred corresponding editors, collaborators and correspondents. Illustrated with chromo-lithographs, engravings and maps, 1888. F. A. Davis, Publisher.

Vols. first and second have been favorably noticed by the JOURNAL. Volumes third, fourth and fifth have been received, and what was said in praise of the first two volumes can be repeated of the remaining three volumes of the series now before us. It is a valuable work, and the editor has well earned the compliments paid him by the whole profession upon his success.

---

ATLAS OF VENEREAL AND SKIN DISEASES. With Original Text; by Prince A. Morrow, A. M., M. D., New York. Wm. Wood & Co., N. Y. Price $2 each.

This publication is to be in fifteen monthly parts—large folio—each part containing five large chromo-lithographic plates.

Five of the parts have already been noticed in this JOURNAL. Numbers 6, 7, 8 and 9 are now before us. These parts came well up in point of excellence to parts first received. The large colored plates, life size, bring the disease as found in practice to the physician in a most comprehensive light. He sees what he is treating. One may gather a fair idea of a disease by reading a description of it, but he never gets a practical conception of it until he sees it. This work is the most thorough and artistic that we have had the pleasure of examining.

The following Transactions of State Medical Associations have been received:

"Transactions of the Maine Medical Association," Vol. IX, Part III, 36th Annual Session, 1888. Portland: Stephen Berry, printer. S. H. Weeks, M. D., Portland, President; S. W. Wakefield, M. D. Warren, Corresponding Secretary.

"Proceedings of the Connecticut Medical Society," 1888. Ninety-seventh Annual Convention, held at New Haven. New Series, Vol. IV, No. 1. Geo. D. Porter, M. D., Bridgeport, President; N. E. Wardin, M. D., Bridgeport, Secretary.

"Proceedings of the Medical Society of Delaware," 1888. Ninety-ninth Annual Session, held at Georgetown. W. T. Skinner, M. D., Pesident; J. A. Ellegood, M. D., Laurel, Secretary.

"Transactions of the Medical and Chirurgical Faculty of the State of Maryland." Ninetieth Annual session, held at Baltimore, 1888. John Morris, M. D., Baltimore, President; J. T. Smith, M. D., Baltimore, Secretary.

"Transactions of the State Medical Society of Tennessee," 1888. Fifty-fifth Annual Session, held at Knoxville. T. J. Happel, M. D., Trenton, President; D. E. Nelson, M. D., Chattanooga, Secretary.

"Transactions of Colorado State Medical Society," 1888. Eighteenth Aunual Session, held at Colorado Springs. S. A. Fisk, M. D., President; H. W. McLaughlin, M. D., Denver, Secretary.

"Transactions of the Medical Society ot New Jersey," 1888. Held at Schooley's Mountain. One Hundred and Twenty-second Annual Session. H. Genet Taylor, M. D., Camden, President; Wm. Elmer, Jr., M. D., Trenton, Secretary.

